# Functional MRI of emotional memory in adolescent depression

**DOI:** 10.1016/j.dcn.2015.12.013

**Published:** 2015-12-31

**Authors:** Rosemary J. Holt, Julia M. Graham, Kirstie J. Whitaker, Cindy C. Hagan, Cinly Ooi, Paul O. Wilkinson, Adrienne O. van Nieuwenhuizen, Belinda R. Lennox, Barbara J. Sahakian, Ian M. Goodyer, Edward T. Bullmore, John Suckling

**Affiliations:** a Department of Psychiatry, University of Cambridge, UK; b Department of Psychiatry, University of Oxford, UK; c Cambridge and Peterborough NHS Foundation Trust, UK; d MRC/Wellcome Trust Behavioural and Clinical Neuroscience Institute, University of Cambridge, UK

**Keywords:** Depression, Adolescence, Memory, fMRI, Emotion

## Abstract

**Introduction:**

Major Depressive Disorder (MDD) is a leading cause of disease burden worldwide. Mood-congruent biases in memory tasks are frequently reported in MDD patients, with facilitated memory for negative stimuli. Most functional MRI studies to date have examined the neural correlates of these biases in depressed adults, with fewer studies in adolescents with MDD. Investigation of MDD in adolescence may aid greater understanding of the aetiology and development of the disorder.

**Methods:**

Cognitive biases were investigated in 56 MDD patients aged 11–17 years and a matched group of 30 healthy control participants with a self-referential memory task. Behavioural performance and BOLD fMRI data were collected during both encoding and retrieval stages.

**Results:**

The neural response to encoding in adolescents with MDD was found to differ significantly from controls. Additionally, neural responses during encoding and retrieval showed differential relationships with age between patient and control groups, specifically in medial, temporal, and prefrontal regions.

**Conclusions:**

These findings suggest that during adolescence neurophysiological activity associated with emotional memory differs in those with depression compared to controls and may be age sensitive.

## Highlights

• Adolescents with Major Depressive Disorder (MDD) and controls were investigated.
• Performance and fMRI data were collected during a self-referential memory task.
• Neural activation during encoding differed between the groups.
• There were differential relationships with age in patients and controls.
• Task related neural activation was related to depression severity in patients.

## 1 Introduction

Early onset Major Depressive Disorder (MDD) is associated with a lifetime prevalence rate of 11% (Avenevoli et al., 2015). However the neural basis of this disorder remains poorly understood particularly during adolescence. In adults disruptions have been noted in cognitive affective biases that are linked to dysfunctional brain activity in prefrontal cortex (PFC), subcortical, and medial temporal regions, including striatum, hippocampus (HC) and amygdala (AMG) (Clark et al., 2009, Roiser and Sahakian, 2013). Cognitive biases occur across a range of processes in MDD and include a greater likelihood of remembering negative over positive material (Leppänen, 2006, Foland-Ross and Gotlib, 2012) as well as greater attentional engagement with dysphoric facial emotion expressions (Gur et al., 1992, Surguladze et al., 2004, Leppänen, 2006, Foland-Ross and Gotlib, 2012).

Affective memory biases, a key element of the cognitive (Disner et al., 2011) and neuropsychological models of MDD (Roiser and Sahakian, 2013), are a common finding (Dalgleish and Werner-Seidler, 2014, Bradley and Mathews, 1988). Dalgleish and Werner-Seidler (2014) highlight the key components of the dysfunction of memory processes seen in depression including biases towards the retrieval of negative material specifically during the recollection of negative personal memories or the false generation of these memories. Recall of positive memories is conversely biased with poor access to these memories or an inability to derive emotional benefit from positive recollections. In a word recall task adult MDD patients remember more negative than positive words, while control participants show the inverse pattern (Bradley and Mathews, 1988).

Functional neuroimaging of emotional memory tasks reveals dysfunction in various brain regions. During encoding of positive words, adult MDD patients have altered activity in anterior cingulate (ACC), PFC, HC and AMG, while during negative encoding there is altered activity in the AMG, ACC and insula (Hamilton and Gotlib, 2008, Arnold et al., 2011, van Tol et al., 2012). These regions of dysfunction are aligned with recent meta-analytic evidence of neural dysfunction in MDD across a range of functional MRI paradigms (Graham et al., 2013), demonstrating that affective memory tasks provide a useful probe for the neural dysfunction observed more broadly in MDD.

There is evidence from juvenile samples that similar biases in memory processing may also be associated with depression in children under 12 years (Jacobs et al., 2008, Bishop et al., 2004, Drummond et al., 2006, Rudolph et al., 1997), and in an adolescent MDD group such biases show a relationship with age, with greater biases for negative information shown in older individuals (Neshat-Doost et al., 1998). In that study a positive correlation between self-reported severity of depression and recall performance also was identified. Changes in affective memory biases with age could have important implications for the presentation of MDD at different ages and the subsequent treatment of the condition. These biases are also present in adolescents with high emotionality (a risk factor for MDD) in the absence of current or past mental illness, suggesting a cognitive bias vulnerability may antedate the emergence of MDD per se (Kelvin et al., 1999, Roiser and Sahakian, 2013). Improving our understanding of the neural substrate of MDD during adolescence, in its emergence, may also shed light on cognitive and neural mechanisms of the disorder that become evident in adult patient populations.

Studies in adolescent MDD support dysfunction in frontal, limbic and occipital areas, in accordance with the adult literature (Halari et al., 2009, Roberson-Nay et al., 2006, Tao et al., 2012, Yang et al., 2009, Yang et al., 2010). Roberson-Nay et al. (2006) report reduced memory for faces and differential activation in the amygdala and anterior hippocampus during memory encoding in adolescents with MDD. Recent reviews also highlight the key areas of functional difference in adolescent MDD, and demonstrate the importance of cortical-limbic deficits shown by lower connectivity in networks associated with emotional dysregulation (Hulvershorn et al., 2012). Furthermore, functional alterations in the ventromedial frontal regions, orbito-frontal and anterior cingulate cortices and subcortical regions including the amygdala and striatum in depressed children appear in both task and non-task (i.e., resting state) acquisitions (Kerestes et al., 2014).

In this study we examine behavioural performance and functional activation on a self-referential word-based memory task in a large sample of adolescents with MDD and healthy controls. Firstly, we consider the behavioural performance for encoding and recognition in adolescents with MDD and controls, considering four outcome measures: categorisation accuracy; categorisation reaction time; memory sensitivity (*d*′); and recognition reaction time. Secondly, considering the neural response to this task we examined the neural correlates of affective memory processing for both encoding and retrieval in adolescents with MDD compared to controls using functional MRI. Subsequently, we aimed to consider age-related effects and group-by-age interactions, in this cross sectional sample, and the association with affective memory biases as a potential marker of developmental influences on the neural systems sub-serving memory encoding and retrieval processes. We also considered the neural correlates of depression symptom severity.

## 2 Materials and methods

### 2.1 Participants

DSM-IV defined MDD male and female patients participated through the IMPACT clinical trial that recruited patients from UK NHS clinics (Goodyer et al., 2011). A diagnosis of current MDD was determined from information gathered from both adolescents and parent interviews using the Kiddie Schedule for Affective Disorders and Schizophrenia–Present and Lifetime version (Kaufman et al., 1997). Standard exclusion criteria for the study included drug or alcohol abuse or dependence, pervasive developmental disorder, presence of learning difficulties, use of medication that may have adverse effects when taken with SSRIs as well as standard MRI exclusion criteria.

Age-, gender-, and handedness-matched healthy control participants (*n* = 34) were recruited from mainstream schools in the local community. Handedness was ascertained using the Edinburgh Handedness Inventory (EHI) (1971) for all participants. Inclusion criteria included no lifetime history of any psychopathology, no mental illness history in the immediate family of an affective disorder or substance dependence, and no current sub-clinical depressive symptoms (<5 on the self-reported Short Mood and Feelings Questionnaire SMFQ, which is well below the clinical cut-off of ≥8 (Angold et al., 1995)). In addition, the exclusion criteria for the MDD group detailed above was also applied to the control group. A larger sample of individuals with current MDD was recruited due to the potential effects of clinical heterogeneity on any results, therefore increasing the power and thus our ability to detect functional differences associated with MDD.

All participants were aged 11–17 years and met MRI safety criteria. All participants provided informed assent or written consent and their parent/legal carer provided written informed consent to be enrolled in the study. The study received ethical permission from the Cambridge Regional Ethics Committee (ref: 09/H0308/168) and appropriate permissions from all relevant NHS trusts where participant recruitment took place.

From 100 patients and 34 healthy controls assessed, 16 patients and four controls were excluded for the following reasons; non-conformity to behavioural task (3 patients, 1 control); brain abnormality (1 patient, 1 control); dental braces (8 patients, 2 controls); failure to complete scan (1 patient); MRI scans took place following the first treatment session (3 patients). Twenty-eight of the patients with MDD were taking SSRIs, these participants were removed from the analyses presented in the main text (results including these individuals are presented in the supplemental materials). The final cohort was constituted of 56 patients (11 male, 45 female) and 30 healthy control participants (6 male, 24 female) (Table S1).

On the day of the MRI assessment, all participants completed the state/trait anxiety inventory (STAI-S: state anxiety; STAI-T: trait anxiety) (Spielberger et al., 1970) and EHI as well as practice tasks ahead of the MRI scan. The entire MRI protocol consisted of structural and functional scanning and lasted approximately 90 min (Hagan et al., 2013). Following the scan, all participants completed the SMFQ to indicate severity of depressive symptoms at the time of scanning. These scores were used in the corresponding analyses rather than those obtained at prior assessments. Further details of the protocol can be seen in the trial publications for the studies (Goodyer et al., 2011, Hagan et al., 2013).

### 2.2 Encode-retrieve fMRI task design

**Fig. 1:**
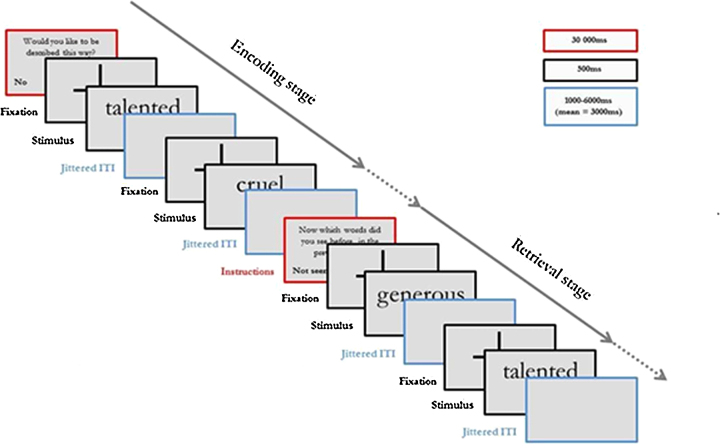
Schematic diagram of trial events in the encode/retrieve task. Boxes are labelled by stage, and box outline indicates duration: red = 30 s, black = 500 ms and blue is a jittered ITI with a mean of 3000 ms (range 1000–6000 ms), events depicted here, in order: encoding instructions, positive categorisation, negative categorisation, retrieval instructions, positive new word recognition (distractor: possible outcomes are correct rejection and false alarm), positive old word recognition (target: possible outcomes are hit and miss). Timeline indicates direction of progression, dashed line indicates where task continues but is not detailed in the diagram. In the retrieval stage, event types not shown are negative new word and negative old word. (For interpretation of the references to color in figure legend, the reader is referred to the web version of the article.)

This task was designed to assess the neural correlates of incidental memory for valenced words. Words were chosen from the Anderson (1968) set of 555 personality trait words. To minimise the ambiguity of the valence of the words selected, we chose words rated for ‘likableness’ (i.e. relating to negative and positive words) in the top and bottom thirds of the word list. Words in each of the four sections of the task were matched for mean word length (negative: *p* = .46; positive *p* = .46) and for frequency of use in the English language (negative: *p* = .47; positive: *p* = .49) (Kucera and Francis, 1967). To help ensure comprehension of the words used in this task in the study age range, the full word list was tested on a group of four 11–12 year olds of average ability to identify any words not recognised or understood in this age group. Encoding and retrieval stages of the memory task ran consecutively (see Fig. 1). Participants viewed the task stimuli projected onto a screen via a mirror fitted to the head coil. A button box in the participant's right hand captured task responses.

In the encoding stage participants completed a categorisation task, indicating whether they would be pleased or upset if they were to be described according to each word presented. Each of the 60 words (30 each of positive and negative valences) were presented, one at a time. At the end of the encoding stage and to introduce the unanticipated retrieval stage, participants viewed an instruction that appeared on the screen for 30 s stating, “And now, which of these words have you seen before, in the previous task? Left (index finger) = Not seen before, Right (Middle finger) = Seen before”.

Trials in the encoding stage were defined by outcome in the retrieval task as hit (subsequently recognised) and miss (subsequently forgotten) separately for each valence. The retrieval stage consisted of all 60 words from the encoding stage (old words) and 60 matched distractors (new words) of which, again, 30 were of positive and 30 were of negative valence. Thus in the retrieval stage there were four possible outcomes for each valence: old word/recognised (hit); old word/not recognised (miss); new word/correctly identified as new (correct rejection); and new word/incorrectly identified as old (false alarm).

In both stages, word trials began with a 500 ms fixation cross, then 500 ms word presentation, followed by a jittered inter-trial interval (ITI) (mean 3000 ms; range 1000–6000 ms) which sampled a Poisson distribution. We recorded button choice and response latency in both stages. The task lasted 12 min 54 s in total. With the exception of the 60 words repeated during the retrieval stage all words were presented only once in an event-related paradigm.

### 2.3 fMRI acquisition

Structural and functional MRI data were obtained at the Wolfson Brain Imaging Centre, University of Cambridge, UK with a Siemens Magnetom Tim Trio scanner operating at three Tesla (3 T). Whole brain echo-planar imaging (EPI) data depicting blood oxygen level dependent (BOLD) contrast was acquired with the following parameters: 32 slices; slice thickness 3 mm plus 0.75 mm interslice gap; field of view 192 mm × 192 mm; 64 × 64 matrix; repetition time 2000 ms; echo time 30 ms; voxel size 3.0 mm × 3.0 mm × 3.0 mm; bandwidth 2442 Hz/pixel; and flip angle 78°. Slices were oriented parallel to the AC-PC line and collected in an interleaved manner. In total, 384 volumes were collected. IR-prepped 3D GRE T1-weighted images of the whole brain using a Magnetisation Prepared Rapid Acquisition Gradient Echo (MPRAGE) sequence permitted subject-specific registration of functional images to standard space.

### 2.4 Analysis of task performance data

Performance in the encoding and recognition sections of the task were analysed separately. In the encoding stage categorisation accuracy and response latency were analysed. To determine memory performance in the retrieval stage of the task, the *d*′ measure of memory sensitivity was calculated. This method, which is based on signal detection theory calculations (Snodgrass and Corwin, 1988), has also been utilised in previous studies of memory performance in depressed youth (Roberson-Nay et al., 2006). Briefly, the number of hits and a false alarms was measured and used to estimate the corresponding *z*-values. Memory sensitivity was then calculated using the formula: *d*′ = z_(false alarm)_ − *z*_(hit)_. This value indicates the subjective salience of old words as distinct from distractor words during the recognition stage.

Behavioural data (categorisation accuracy; categorisation reaction time; memory sensitivity (*d*′); and recognition reaction time) were analysed using a repeated measures ANCOVA (SPSS inc, v17), with valence as a within-subject factor, group as a between-subject factor and age included as a covariate. Accuracy was calculated from the total number of events responded to, excluding events where no response was made due to the short time interval in which participants could respond.

### 2.5 Analysis of fMRI data

#### 2.5.1 Image processing

Functional MRI data were analysed with FEAT Version 6.0 (FMRIB's Software Library [FSL], http://www.fmrib.ox.ac.uk/fsl). We analysed encoding and retrieval stages separately following identical image pre-processing: The first six EPI volumes were discarded and non-brain material identified and removed using the Brain Extraction Tool (BET) of FSL (Smith, 2002). A correction for head motion was applied and then each 3D volume was spatially smoothed using a Gaussian kernel of 6 mm full-width at half-maximum. Once complete the 4D acquisition was grand mean intensity normalised, slice-time corrected with Fourier-space time-series phase-shifting before high-pass temporal filtering (90 s). EPI images were co-registered to standard anatomical space (Montreal Neurological Institute 152 stereotactic atlas; 2 mm × 2 mm × 2 mm) via the subject-specific high-resolution image (MPRAGE) using the FLIRT tool, and then further refined using the non-linear registration technique FNIRT (Jenkinson and Smith, 2001, Jenkinson et al., 2002).

Two separate event-related designs were used to investigate the neural correlates of successful encoding and retrieval separately using within-subject, fixed effects analyses. Events were modelled as occurring at word presentation and convolved with a gamma haemodynamic response function.

#### 2.5.2 Encoding stage

In the encode stage, the contrast of words subsequently remembered (hits) with those subsequently forgotten (misses) gave the neural correlates of *successful encoding* for each valence. Additionally, the contrast of all negative against all positive trials gave a proxy of *affective bias in encoding* and the contrast all positive and negative words against baseline gave the neural correlates of *encoding attempt*.

#### 2.5.3 Retrieval stage

In the retrieve stage, the contrast of hits (recognising an old word) against correct rejections (correctly identifying a new word as such) gave the neural correlates of *successful retrieval* for each valence. Additionally, the contrast of negative against positive trials gave a proxy for *affective bias in retrieval* and the contrast of all positive and negative words against baseline gave the neural correlates of *retrieval attempt*. A similar analysis strategy for the behavioural and fMRI data was implemented in Miskowiak et al. (2007).

#### 2.5.4 Statistical models

For all the higher level analyses in both encoding and retrieval stages of the task, random effects analysis identified regions elicited by the tasks averaged across all participants. This was thresholded using cluster determined by *Z* > 2.3 and a (corrected) cluster significance threshold of *p* = 0.05 (Worsley, 2001).

Following this a between-subjects, random effects design was used to test for the main effects of group and age and the group-by-age interaction in a whole brain analysis. Finally, within the patient group only we undertook a whole-brain analysis to investigate where BOLD activation might be significantly related to depression severity (denoted by higher SMFQ score). Results were thresholded with a cluster level threshold of *p* = 0.05.

For the main analyses, patients taking SSRIs were excluded. This step was taken in order to reduce differences in neurological responses due to medication rather than then pathological effects of depression. This approach is supported by previous literature which provides evidence for functional neural differences following SSRI administration (Rizvi et al., 2013, Roy et al., 2010, Walsh et al., 2007). This sub-group may therefore introduce additional variance into the analyses. All analyses were repeated including the full cohort of patients including those taking SSRIs with results presented in the supplementary material, demonstrating the robustness of the results in the wider cohort and reducing the risk of type two error. Finally, all unthresholded statistical maps were uploaded to NeuroVault at http://neurovault.org/collections/1015.

## 3 Results

### 3.1 Behavioural performance

Overall average accuracy was high in both tasks (categorisation: 91%; recognition: 74%) in line with that observed in a similar task in healthy adults (Miskowiak et al., 2007). Behavioural data and results of ANCOVA tests are presented in Table 1. Of note, groups did not differ in their accuracy of categorisation during encoding, memory sensitivity during retrieval or response latency to positive or negative words. There was a main effect of age on reaction time for encoding and retrieval such that older participants had faster reaction times (Encoding: *F* = 9.50; *p* = 0.003; Retrieval: *F* = 8.421, *p* = 0.005). During retrieval there was also a main effect of valence (*F* = 7.196; *p* = 0.009), a main effect of age (*F* = 4.063; *p* = 0.047) and a valence-by-age interaction for the *d*′ score of memory sensitivity (*F* = 6.566; *p* = 0.012). Participants were more likely to remember negative words and this pattern increased with age. Results including the patients taking SSRI medications are described in the supplementary materials.

**Table 1:** Behavioural performance measures in patients (excluding those taking antidepressant medications) and control groups during encoding (categorisation) and retrieval (recognition) stages of the encode/retrieve task. Statistical results of ANOVA model including patient group (patients/controls) and valence (negative/positive) in a repeated measures design, with participant age included as a covariate in the model. Bold text indicates that result was significant at the *p* < .05 level.

Task Measure	Depression (*n* = 56)		Healthy control (*n* = 30)		Analysis													
	Mean	S.D.	Mean	S.D.	Main effect of valence		Main effect of group		Group × valence interaction		Main effect of age		Group × age interaction		Valence × age interaction		Group × valence × age interaction	
					*F*	*p*	*F*	*p*	*F*	*p*	*F*	*p*	*F*	*p*	*F*	*p*	*F*	*p*
*Emotional categorisation*																		
Accuracy																		
Negative	0.894	0.127	0.932	0.066	0.029	.855	0.032	.859	0.098	.755	2.472	.120	0.088	.768	0.020	.887	.108	.743
Positive	0.892	0.147	0.927	0.072														
Reaction time (ms)																		
Negative	1329.5	281.4	1369.7	265.07	2.42	.123	.471	.494	0.775	.381	**9.50**	**.003**	0.386	.536	1.834	.179	0.745	.390
Positive	1291.8	270.7	1339.5	300.74														

*Emotional recognition*																		
Memory sensitivity (*d*′)																		
Negative	1.517	0.681	1.483	0.557	**7.196**	**.009**	0.749	.781	0.379	.540	**4.063**	**.047**	0.081	.776	**6.566**	**.012**	.303	.584
Positive	1.376	0.746	1.443	0.640														
Reaction time (ms)																		
Negative	1358.0	259.7	1478.8	300.63	0.007	.932	2.222	.140	0.546	.462	**8.421**	**.005**	1.756	.189	0.274	.602	0.629	.430
Positive	1282.7	228.1	1383.0	283.9														

### 3.2 FMRI results: Encoding

*Successful encoding* (hits > misses) of words activated a broad network of regions including frontal, temporal, and occipital regions with both groups combined (*Z* = 6.16, 13940 voxels, [−44, 38, −8]). Examining the whole cohort, no participants had zero misses and the mean number of missed trials was 14.74 (SD = 7.074) (positive words: *M* = 6.36, SD = 3.82; negative words *M* = 8.38, SD = 4.09), the distribution of scores is demonstrated in Fig. S1.

**Fig. 2:**
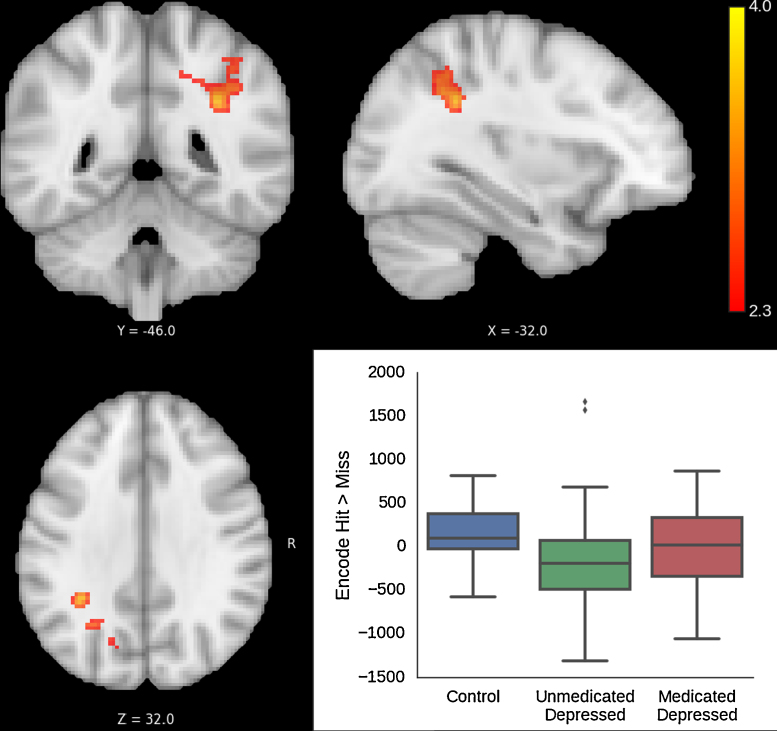
BOLD signal during successful encoding (hit > miss) of positive and negative words where greater activation was identified in controls compared to the depressed group (excluding patients taking antidepressant medications). Patients taking medications are included in the plot for illustrative purposes only. The 3D unthresholded statistical map can be found at http://neurovault.org/images/14547.

**Fig. 3:**
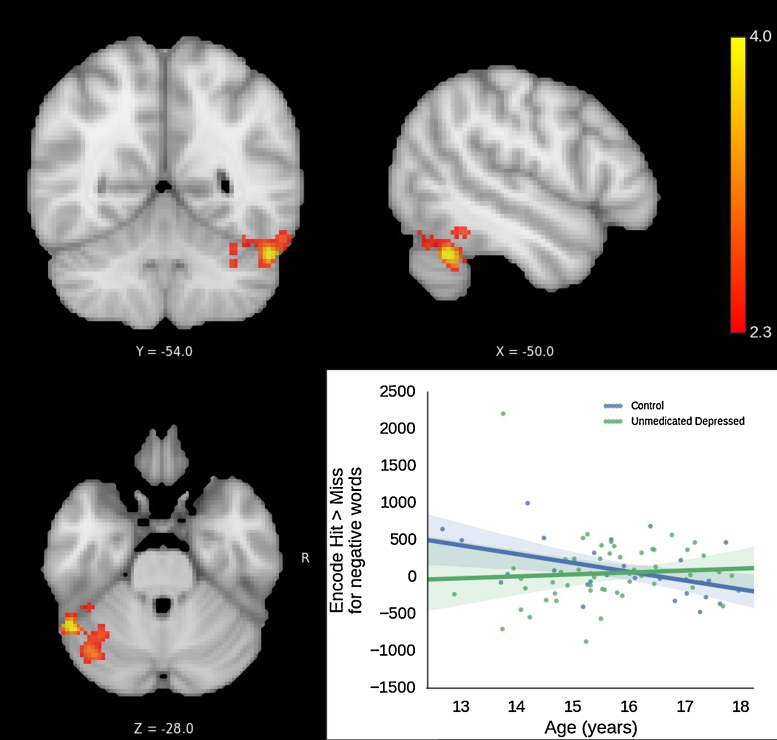
BOLD signal during successful encoding of negative words (hit > miss) where a significant group by age interaction was identified (excluding patients taking antidepressant medications). Scatter plot illustrates association with age in controls and depressed patients.

There was a significant between-group difference such that the depressed group showed larger, but in the opposite direction, differences in activation for the contrast of hits > misses of positive and negative words combined. Differences in activation were located in the left supramarginal gyrus, lateral occipital cortex and superior parietal lobe (Fig. 2, Table S2). A negative relationship between cross sectional age and successful encoding of negative words was evident including activation in the left frontal pole and superior frontal gyrus (Table S3, Fig. S2). Additionally there was a significant group-by-age interaction for the successful encoding of negative words, with differential activation located in regions including the occipital cortex, fusiform gyrus and inferior temporal gyrus (*z* = 4.26, 1058 voxels, [−50, −54, −28]) (Fig. 3). The direction of this interaction was such that there was a stronger negative association with age in the control group compared to patients. There was no significant association between activation and depression severity.

### 3.3 Affective bias in encoding (all negative > all positive)

Activation for the contrast of negative > positive words across all participants did not reach significance. However, the contrast of positive > negative words across all participants resulted in activation in the right frontal pole (Z = 3.74, 1283 voxels, [48, 54, 4]).

The higher level analyses of group, age and correlation with symptom severity for the affective bias contrast did not reach significance. Significant effects were identified in the larger sample of depressed patients (including those taking SSRIs), where a significant association with age and depression severity was identified. Further details are given in the supplementary material.

### 3.4 Encoding attempt (all negative and positive words > baseline)

**Fig. 4:**
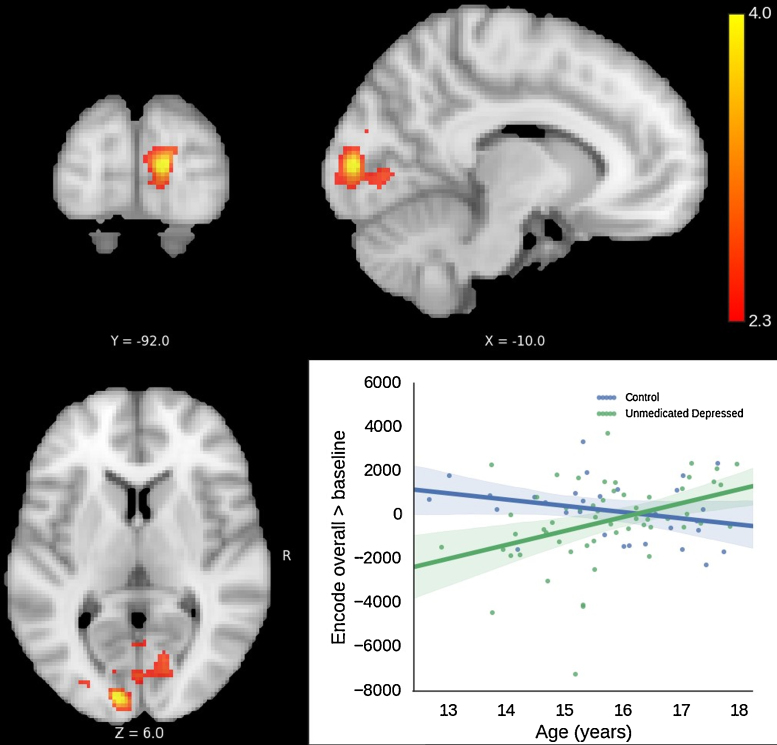
BOLD activation during encoding attempt (all words > baseline) where a significant group by age interaction was identified. (This group by age interaction was only evident when excluding the patients taking antidepressant medications.) The 3D unthresholded statistical map can be found at http://neurovault.org/images/14644.

The contrast for encoding attempt across all participants activated four clusters (peak cluster: *Z* = 6.46, 12077 voxels, [−4, 14, 44], including cingulate, paracingulate, pre and post central gyrus and insular). When comparing the patients and controls, there was no significant difference between groups. There was a significant positive association with cross sectional age identified in the middle temporal gyrus, supramarginal gyrus and occipital pole (*Z* = 3.97, *p* = 0.009, 1001 voxels, [−68, −54, 12]). Additionally there was a whole brain group-by-age interaction identified in two clusters (occipital pole, lateral occipital cortex; *Z* = 4.41, *p* = 0.0024, 1239 voxels [−10, −92, 6]): supramarginal, postcental and precentral gyrus; *Z* = 3.92, *p* = 0.0283, 803 voxels, [52, −28, 46]. The direction of this interaction was such that the relationship between age and brain activation was stronger in the patient than in the control group (Fig. 4).

### 3.5 FMRI results: Retrieval

#### 3.5.1 Successful retrieval (hits > correct rejections)

The effects of this contrast across groups and across negative and positive words activated the fronto-parietal network, cingulate and occipital regions and led to deactivation of frontal, temporal and lateral occipital regions (*Z* = 8.65, 9543 voxels, [−38, −54, 46]).

**Fig. 5:**
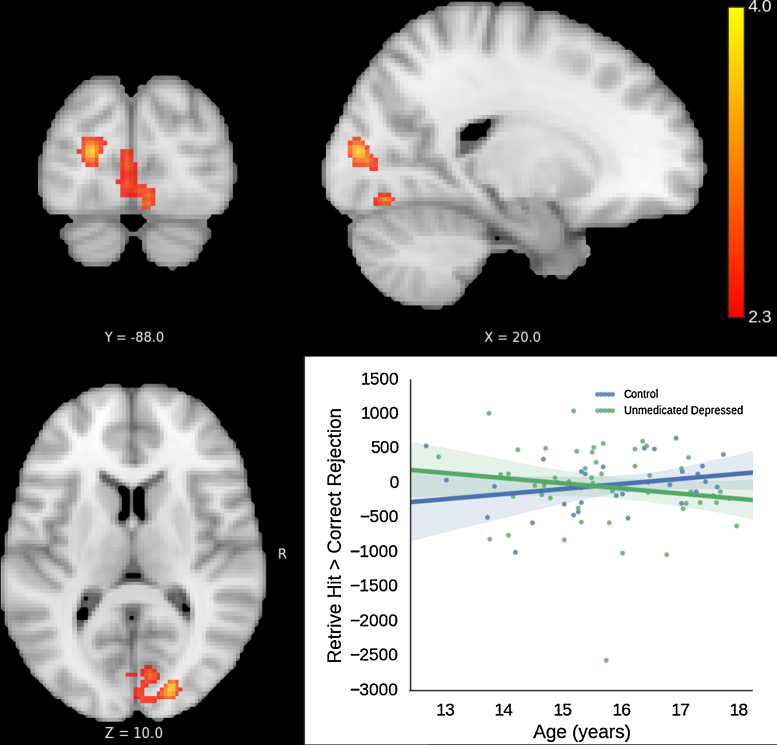
BOLD signal during retrieval attempt (hit > correct rejection) showing a group by age interaction (when excluding patients taking antidepressant medications). Scatter plot illustrates the group by age interaction in the two groups. The 3D unthresholded statistical map can be found at http://neurovault.org/images/14714.

Higher level analyses did not identify a case–control difference or a significant association with age. However, a significant group-by-age interaction was evident located in the primary visual cortex, occipital pole and lingual gyrus. For this contrast the control group showed a stronger positive association between activation and age compared to the patient group (Table S4, Fig. 5).

#### 3.5.2 Affective bias in retrieval (all negative > all positive trials)

The effect of this contrast across groups activated a network of regions including the superior parietal, frontal and occipital regions (peak cluster; *z* = 6.14, 31358 voxels, [−34, −56, 44]). In all participants, the affective bias in retrieval (all negative > all positive trials) revealed a positive association with age in four clusters including the frontal and occipital regions (Table S5). In both groups, the affective bias contrast was, on average, negative in the younger participants indicating relatively greater activity in response to positive material. There were no significant group differences, group-by-age interactions or correlation with depression severity.

#### 3.5.3 Retrieval attempt (all retrieval trials  > baseline)

**Fig. 6:**
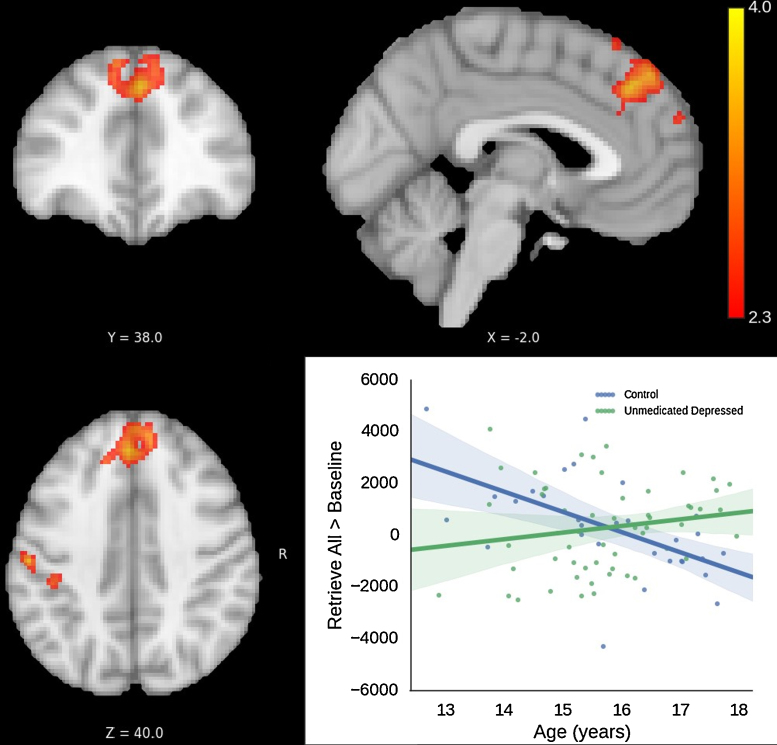
BOLD activation during retrieval attempt (all retrieval trails > baseline) where a significant group by age interaction was identified (excluding patients taking antidepressant medications). The 3D unthresholded statistical map can be found at http://neurovault.org/images/14798.

**Fig. 7:**
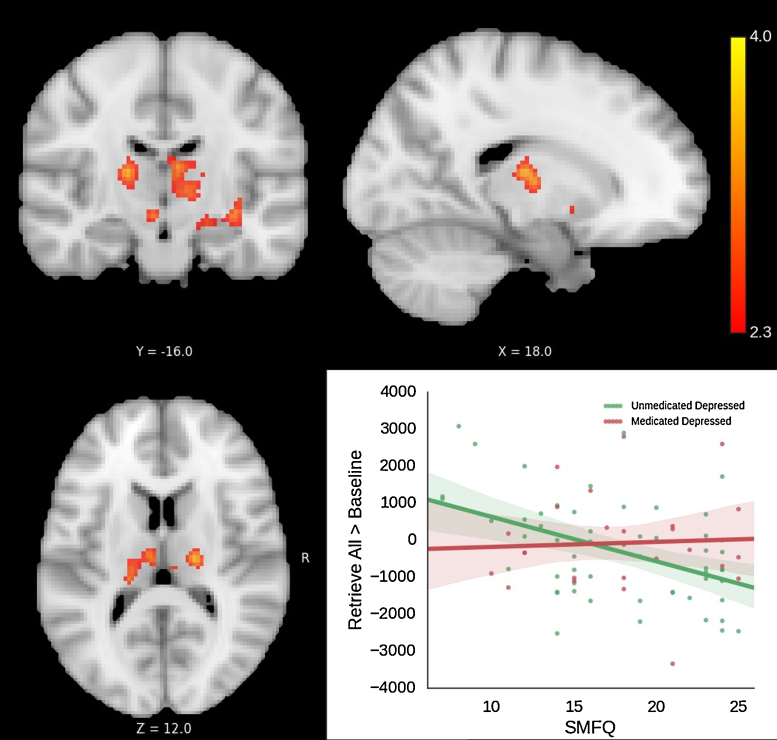
BOLD activation during retrieval attempt (all retrieval trails > baseline) showing a significant correlation with SMFQ in the depression group (not taking antidepressant medications). The medicated group is included in the scatter plot for illustrative purposes only. The 3D unthresholded statistical map can be found at http://neurovault.org/images/14795.

The effect of this contrast across groups activated a network of regions including the Para-cingulate, cingulate gyrus and postcentral gyrus (*Z* = 5.5, 2231 voxels, [6, 14, 50]). The patient and control groups did not differ significantly, however there was a significant negative association with age across groups (*Z* = 4.16, 1686 voxels, [26, −20, 44]) located in regions including the precentral gyrus (Fig. S3). There was also a group-by-age interaction (*Z* = 3.58, 913 voxels, [−2, 38, 40]) located in superior frontal and precentral gyrus (Fig. 6; Table S6). The direction of this interaction was such that the decrease in activation with cross-sectional age was more pronounced in the control group compared to the depressed group. A significant negative correlation between activation during retrieval attempt and depression severity was also identified, located bilaterally in the thalamus (*Z* = 3.42, 1069 voxels, [18, −16, 12]) (Fig. 7).

## 4 Discussion

In this fMRI study of affective memory processing in adolescent MDD, we found whole-brain case–control differences in neural activation during encoding attempts of affective word stimuli. A main effect of age and a group-by-age interaction were also identified for successful encoding of negative words. For memory retrieval there were no whole brain case–control differences, however an association with age was identified during affective bias and retrieval attempt. Group-by-age interactions were also identified during successful retrieval and retrieval attempt.

Behavioural changes in reaction times for emotional categorisation and emotional recognition of both positive and negative material increased with age across both groups, however no case–control differences were identified. This is inconsistent with some previous literature identifying case–control differences in the affective bias of memory processing (Dalgleish and Werner-Seidler, 2014, Bradley and Mathews, 1988). However the current findings may be consistent with reports of age related changes in memory performance (Neshat-Doost et al., 1998).

In line with these findings, BOLD activity during encoding and retrieval stages were also found to be associated with age. This indicates a possible developmental aspect to the behavioural performance and neural activation during this task. Group differences were also identified during the encoding stage of the task, but were not seen in retrieval. Differential activation during successful encoding and encoding attempt was identified in regions including the occipital cortex and cingulate gyrus. This finding suggests that depressed youth may have a differing neural response to encoding of emotional state words. This finding is consistent with previous studies in adults (Hamilton and Gotlib, 2008, Arnold et al., 2011, van Tol et al., 2012) and adolescents (Roberson-Nay et al., 2006) with MDD which have identified case–control differences in comparable brain regions during memory encoding. Difference in the structure and function of the cingulate, in particular, has been consistently implicated in depression (Clark et al., 2009). Here we also report reduced activation in the supramarginal gyrus, lateral occipital and superior parietal cortex, which have been implicated in language and visual processing. Previous fMRI studies have focused on the encoding stage of this task rather than on retrieval, and hence further work is needed to determine if group differences are in fact characteristic during this aspect of memory in MDD.

In the current study we also identified group-by-age interactions during memory encoding located in clusters including occipital cortex, pre and post central gyrus and inferior temporal regions. These interactions were not significant when including depressed patients taking antidepressant medications. The different relationships with age suggest that in the depressed group there may be changes in typical development, contrary to normal maturation and that these relationships may become normalised through the use of anti-depressant medication. Longitudinal data are required to fully test these hypotheses as the current study comprised a cross sectional sample. Prospectively following a healthy cohort, some of whom become depressed and then recover, would aid the understanding of any aberrant developmental processes in adolescent depression.

In the current study, MDD self-report symptom scores indexing episode severity were linked with altered neural activity during retrieval attempt. This is consistent with reports of dysfunction in the thalamus in previous reports of the neural effects of MDD (Graham et al., 2013). When considering the wider cohort (including patients taking SSRIs) there was also an association between depression severity and the neural response to affective bias for encoding identified in frontal regions. This finding is consistent with literature describing frontal lobe dysfunction in MDD (Clark et al., 2009, Roiser and Sahakian, 2013, Kerestes et al., 2014). These findings suggest an association between differing neural activation in these regions and severity of depressive symptomology. Further work is needed for a more detailed examination of the relationship between neural activation with symptom severity and other characteristics such as duration and the number of depressive episodes.

Adolescence appears to be a crucial period in the emergence and aetiology of MDD. It has been proposed that aberrant developmental processes themselves may play a key role in the onset of disorder (Goodyer, 2008, Paus et al., 2008). Further work is required to specifically test this hypothesis. Investigation of grey matter volume in this cohort also revealed the presence of age-related differences in ACC and thalamus (Hagan et al., 2015), emphasising the need for further investigation of MDD across this period of neural development. Such investigations would benefit from including factors that may influence the typically developing brain, including measures of childhood adversity that may provide valuable explanatory power regarding the differences we have observed in adolescents (Dannlowski et al., 2012, Teicher and Samson, 2013). Indeed exposure to an adverse family environment in childhood carrying a moderate to severe risk of childhood maltreatment increases the liability for emerging adolescent depression by 14 years of age by 3–5 times (Dunn et al., 2011). Furthermore, proof of principle findings have suggested that childhood adversities may exert effects on neural systems once past and current mental state factors are taken into account (Walsh et al., 2014) and may indicate a mechanism for atypical neuro-cognitive development. Inclusion of such measures would enable testing of the neurodevelopmental model and determine the contribution of such experience to neural scars in adult life.

### 4.1 Limitations

The task design used in the current study differs from previous studies of memory in individuals with depression. In order to facilitate fMRI data to be collected during both encoding and retrieval stages of the task, a scanner-compatible recognition task was necessary. Although this greatly increased our coverage of memory processing, there is evidence to suggest that affective biases in memory processing in MDD may be better observed in adolescent samples when a free recall task is deployed (Neshat-Doost et al., 1998). However, free recall techniques are difficult to conduct in imaging studies where minimal head movement is permitted. This difference in the task construction may account for the absence of performance differences in memory retrieval presented here. Furthermore other components of memory (e.g. autobiographical memory recall) may be impaired in adolescent MDD and were not tested here. Thus it is possible that our conclusions may have been different had we used a different task design. Future work could incorporate post scan rating of the words by participants to ensure that patients perceive the valence of the word (positive and negative) in the same way as controls. Future paradigms could also include neutral words to enable the comparison of both positive and negative words against a neutral baseline.

A potential caveat of the current study is that some participants have missing fMRI data in the superior parietal lobe as shown in Fig. S4. Coverage in the temporal lobe was prioritised during data collection due to the theoretical implications of activation in this brain region during the task. This missing data may have occurred due to participant movement and slice prescriptions not being adjusted between scans. The findings should therefore be interpreted in accordance with the caveat of limited brain coverage in a proportion of the sample.

Finally, we did not match for socio-economic status in this study and IQ measures were only collected in a subset of the cohort so results should also be interpreted in accordance with this caveat. The cross-sectional age related effects reported here require further investigation with longitudinal designs to confirm the presence of differences in the developmental trajectories of the neural response to memory encoding and retrieval.

In summary; this study investigated the neural correlates of encoding and retrieval of valenced word stimuli in an adolescent MDD group and age, handedness, and sex-matched healthy controls. We identified case–control differences in the neural response to memory encoding, as predicted by the adult literature. However, there were no group effects in the behavioural performance. We also identified associations with age and differential relationships with age in the depressed adolescents compared to controls during both memory encoding and retrieval.

## Conflict of interest

ETB is employed half time by the University of Cambridge and half time by GlaxoSmithKline plc. BJS consults for Cambridge Cognition, Servier, Otsuka, Peak (Brainbow) and Lundbeck; she holds a grant from Janssen/J&J. The other authors report no biomedical financial interests or conflicts of interest.
