# Supplementary material for: Functional MRI of emotional memory in adolescent depression
Source: Dev Cogn Neurosci. 2015 Dec 31;19:31–41. doi: 10.1016/j.dcn.2015.12.013 (PMC4913558; doi:10.1016/j.dcn.2015.12.013)
Supplement: Supplementary file 1 [file mmc1.docx]

**Supplementary Figures**

|  | **Medicated patients (N=28)** | **Un-medicated patients (n=56)** | **Healthy controls (n=30)** | **Controls vs. un-medicated patients** | **Controls vs. patients** | **Medicated vs un-medicated depressed** |
| --- | --- | --- | --- | --- | --- | --- |
| Age | 15.79 (1.14) | 15.69 (1.17) | 15.76 (1.39) | t=-.246 p=.806 | **T=.134, p=.894** | **T=3.42, p=.73** |
| Left: Right | 7:21 | 3:53 | 2:28 | Fishers exact p=1 | **Fishers exact p=.729** | **Fishers exact p=.014*** |
| Male: female | 9:19 | 11:45 | 6:24 | Fishers exact p=1 | **Fishers exact p=.802** | **Fishers exact p=.277** |
| Depression severity | 17.93 (4.55) | 17.63 (5.13) | 2.77 (1.92) | T=19.301, p<.001* | **T=16.189, p<.001*** | **T=.265, p=.79** |
| State anxiety | 46.46 (10.43) | 45.20 (10.15) | 29.13 (6.56) | T=9.88, p<.001* | **T=8.254, p<.001*** | **T=.535, p=.59** |
| Trait Anxiety | 61.36 (7.78) | 59.61 (8.44) | 31.00 (6.57) | T=16.12, p<.001* | **T=17.532, p<.001*** | **T=.919, p=.361** |

**Table S1** Demographic information displayed for patients and healthy controls included in the scanning session. Depression severity measured by SMFQ (Short Moods and Feelings Questionnaire (Angold, et al., 1995)). Anxiety measured by the STAI (State-Trait Anxiety Inventory (Spielberger, 1970), which provides state (STAI-S) and trait (STAI-T) estimates). Age, and estimates of depression and anxiety are shown as “mean (standard deviation)”. *indicates where groups are significantly different (p<0.05).


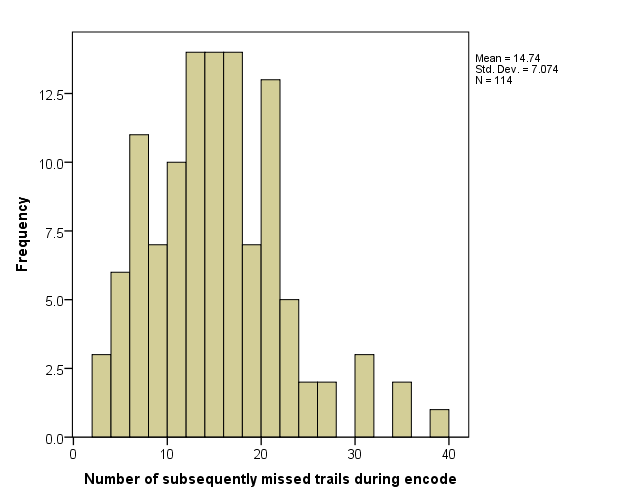


**Figure S1**: Histogram demonstrating the number of subsequently forgotten (referred to as a miss in the encoding fMRI analysis) events included in the fMRI contrast of hits vs misses during encoding.

| Region | Size (voxels) | Z-value | Coordinates | | |
| --- | --- | --- | --- | --- | --- |
|  |  |  | x | y | z |
|  | |  |  |  |  |
| L supramarginal gyrus | 700 | 3.48 | -32 | -46 | 32 |
| L lateral occipital cortex |  | 3.47 | -16 | -66 | 40 |
| L supramarginal gyrus |  | 3.45 | -36 | -50 | 42 |
| L superior parietal |  | 3.30 | -26 | -54 | 44 |
| L lateral occipital cortex |  | 3.22 | -20 | -58 | 42 |
| L superior parietal |  | 3.20 | -30 | -52 | 36 |

**Table S2:** Local maxima of BOLD signal during successful encoding (activations: hit>miss) of negative and positive personality trait words in patients (excluding those taking antidepressant medications) compared to controls (Control > Depressed), thresholded at P=.05.

| Region | Size (voxels) | Z-value | Coordinates | | |
| --- | --- | --- | --- | --- | --- |
|  |  |  | x | y | z |
|  | |  |  |  |  |
| R superior frontal gyrus | 1021 | 3.54 | 2 | 56 | 30 |
| L Frontal Pole |  | 3.47 | -12 | 42 | 52 |
| R Frontal Pole |  | 3.46 | 18 | 64 | 18 |
| R Frontal Pole |  | 3.37 | 2 | 60 | 28 |
| R Frontal Pole |  | 3.27 | 12 | 64 | 16 |
| L Frontal Pole |  | 3.21 | -12 | 52 | 46 |

**Table S3:** Local maxima of BOLD signal during successful encoding of negative words (Hit>Miss) significantly associated with age in patients not taking antidepressant medications and controls, thresholded at P=.05


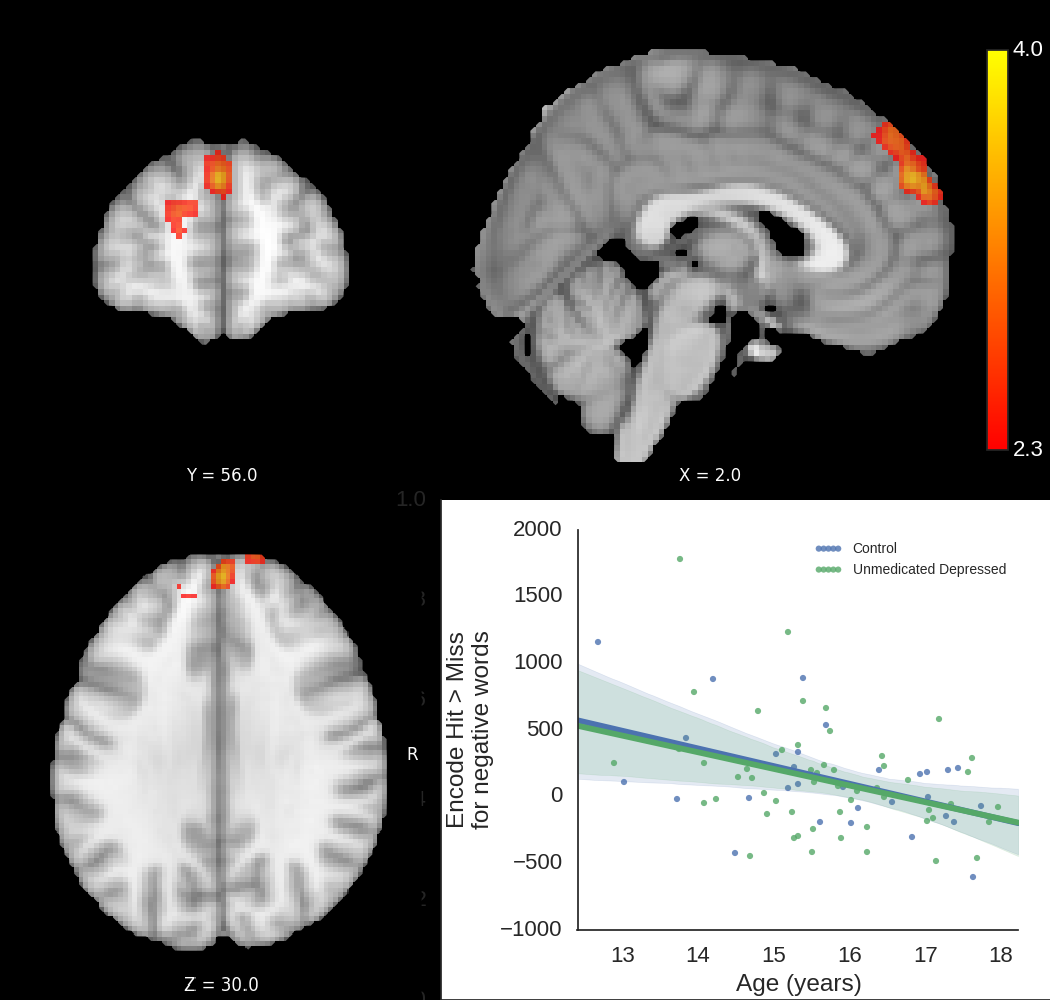


**Figure S2**: BOLD signal during successful encoding of negative words (Hit>miss) showing a significant negative association with age across groups (including only un-medicated depressed patients). Scatter plot illustrates association with age in controls and depressed patients.

| Region | Size (voxels) | Z-value | Coordinates | | |
| --- | --- | --- | --- | --- | --- |
|  |  |  | x | y | z |
|  | |  |  |  |  |
| R Occipital pole | 854 | 3.65 | 20 | -88 | 10 |
| R Intracalcarine cortex |  | 3.39 | 10 | -80 | 16 |
| L Lingual gyrus |  | 3.26 | -6 | -84 | -14 |
| R Occipital fusiform gyrus |  | 3.25 | 20 | -76 | -12 |
| R Occipital pole |  | 3.19 | 6 | -96 | 0 |
| R Intracalcarine cortex |  | 2.96 | 4 | -86 | 4 |

**Table S4:** Local maxima of BOLD during successful retrieval (hit>correct rejection) where a significant group by age interaction was identified (excluding patients taking antidepressant medications), thresholded at P=.05.

| Region | Size (voxels) | Z-value | Coordinates | | |
| --- | --- | --- | --- | --- | --- |
|  |  |  | x | y | z |
|  | |  |  |  |  |
| R Frontal pole | 2884 | 4.21 | 34 | 34 | -12 |
| R Parahippocampul gyrus |  | 3.95 | 18 | -4 | -20 |
| R Frontal Pole |  | 3.94 | 48 | 38 | -6 |
| R Superior temporal gyrus |  | 3.88 | 44 | 22 | -10 |
| R Amygdala |  | 3.83 | 22 | -4 | -22 |
| R thalamus |  | 3.78 | 8 | -28 | 2 |
| L Lateral occipital cortex  L occipital pole  L occipital pole  L occipital Pole  L occipital Pole  L occipital Pole  L Amygdala  R Brain stem  R Brain stem  L Parahippocampul gyrus  L Frontal orbital cortex  L Parahippocampul gyrus  R Lateral occipital cortex  R Lingual gyrus  R Lingual gyrus  R Lateral occipital cortex  R Lateral occipital cortex  R Lateral occipital cortex | 2241  1222  963 | 4.56  3.44  3.42  3.41  3.38  3.27  4.27  3.97  3.91  3.57  3.4  3.38  4.1  3.48  3.26  3.09  3.01  2.99 | -22  -24  -26  --10  -14  0  -20  12  10  -12  -20  -10  38  18  20  36  40  46 | -84  -92  -92  -102  -98  -80  -6  -22  -26  0  6  -12  -82  -70  -64  -86  -70  -72 | 34  14  10  6  14  8  -22  -26  -26  -18  -16  -22  -8  -8  -10  0  -6  -2 |
|  |  |  |  |  |  |

**Table S5:** Local maxima of BOLD for the affective bias during retrieval (Negative> positive) where a significant association with age was identified across both groups combined (excluding patients taking antidepressant medications).

**
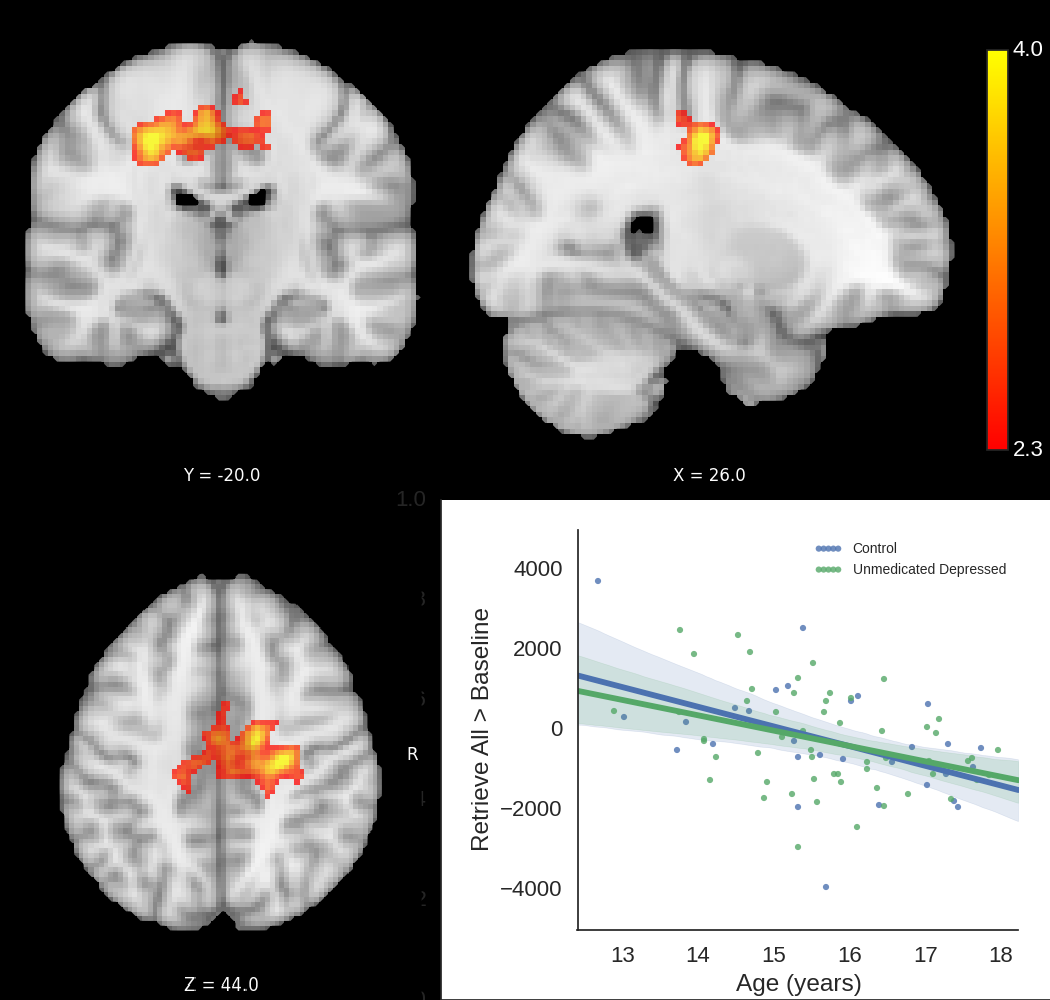
**

**Figure S3**: BOLD activation during retrieval attempt (all retrieval trails > baseline) showing a significant association with age (excluding patients taking antidepressant medications).

| Region | Size (voxels) | Z-value | Coordinates | | |
| --- | --- | --- | --- | --- | --- |
|  |  |  | x | y | z |
|  | |  |  |  |  |
| L Superior frontal gyrus | 913 | 3.58 | -2 | 38 | 40 |
| L Superior frontal gyrus |  | 3.45 | -14 | 34 | 44 |
| Superior frontal gyrus |  | 3.40 | 0 | 46 | 44 |
| R Superior frontal gyrus |  | 3.16 | 8 | 46 | 40 |
| R Superior Frontal gyrus |  | 3.14 | 10 | 42 | 40 |
| R Cingulate gyrus |  | 3.12 | 8 | 18 | 24 |
| R Putamen  R Caudate  R Caudate  L Pallidum  L Caudate  L Putamen  L Postcentral gyrus  L Postcentral gyrus  L Supramarginal gyrus  L Postcentral gyrus  L Postcentral gyrus  L Postcentral gyrus | 830  777 | 3.79  3.47  3.38  3.09  3.08  3.07  3.59  3.56  3.24  3.19  3.14  3.08 | 20  10  6  -20  -6  -26  -40  -54  -44  -42  -60  -58 | 8  0  4  -4  6  0  -30  -20  -32  -28  -24  -14 | 12  12  8  4  10  0  54  40  36  36  59  50 |
|  |  |  |  |  |  |

**Table S6:** Local maxima of BOLD for the contrast of retrieval attempt (all retrieval trails > baseline) where a significant group by age interaction was identified (excluding participants taking antidepressant medications).

*Supplementary results*

The data were re-analysed in independent analyses using the same models as described in the main text. However a broader group of participants were included in the analysis to demonstrate the results for the larger cohort, which included patients taking SSRI medications. 84 patients with MDD (including 56 un-medicated and 28 medicated) and 30 healthy control participants were included in the analyses. The patients taking antidepressant medication and those not taking medications did not differ significantly on any of the demographic measures collected with the exception of handedness.

*Behavioural data*

Repeating these analyses, including patients taking SSRI medications, the results were comparable to those described in the main text. There was a main effect of age on reaction time for encoding and retrieval such that older participants had faster reaction times (Encoding: F=10.08; p=0.002; Retrieval: F=10.22, p=0.002). During retrieval there was also a main effect of valence (F=7.704; p=0.006) and a valence-by-age interaction for the d’ score of memory sensitivity (F=6.866; p=0.010). However the main effect of age for memory sensitivity during retrieval no longer reached significance (F=2.628; p=.18).

*Successful encoding (Hits> misses)*

The whole brain effect for this contrast across both groups activated a similar network as described for the analysis excluding patients taking SSRIs (Z=6.71, 18963 voxels, [-44, 38, -6]). There were no whole-brain between-group differences for this contrast. In all participants, activity in two clusters during successful encoding of negative words (hits > misses for negatively valenced words) decreased significantly with increasing age. This included firstly the left lingual gyrus, parahippocampul gyrus and thalamus (Z=4.14, 1424 voxels, -10, -50, -8). And secondly the bilateral frontal pole, paracingulate gyrus, superior frontal gyrus, and paracingulate gyrus (Z=3.73, 956 voxels, [-12, 50, 14]). There was no significant age effect for successful encoding of positive words or when combining both valences of words. There were also no significant group-by-age interactions.

*Affective bias in encoding (all negative > all positive)*

Activation for the contrast of negative > positive words across all participants did not reach significance. However, the contrast of positive > negative words across all participants resulted in activation in the right frontal pole (Z=4.19, 1870 voxels, [24, 62, 8]).

There were no significant whole-brain case-control differences for affective bias. Across all participants there was a significant positive association between activation and age (Z=4.18, 2365 voxels, [-16, -20, 44]). The brain regions involved in this association included occipital, superior parietal and temporal regions. No significant group-by-age interaction was identified.

A whole-brain analysis was performed in the patient group, correlating BOLD activity for the affective bias contrast during encoding with depression severity scores. Regions in the frontal pole and middle frontal gyrus were found to be significant. The direction of this result was such that the discrepancy between negative and positive trials decreased with increasing SMFQ score (p=0.016, Z=3.53, 798 voxels, [-18, 60, 20]).

*Encoding attempt (all negative and positive works > baseline)*

The contrast for encoding attempt across all participants activated four clusters (peak cluster: Z=8.02, 22311 voxels, p<0.001, [-4, 14, 46]). Between groups analysis of the neural correlates of encoding attempt revealed significant whole-brain, case-control differences showing greater activation in the depressed group compared to controls. Activation differences were located in the precuneous, cingulate gyrus and lateral occipital cortex (Z=3.65, 832 voxels, [-10, -66, 38]). There were no significant correlations of BOLD activity during encoding attempt with age, no significant group-by-age interactions or associations with depression severity.

*Successful retrieval (hits> correct rejections)*

The whole brain activation of this contrast across groups included a similar network to that of the analysis excluding medicated patients (Z=10.1, 12949 voxels, [-40, -56, 46]). There were no significant whole-brain between-group differences for this contrast. In addition, there were no associations with participant age, group-by-age interactions or correlations with depression severity.

*Affective bias in retrieval (all negative > all positive)*

The whole brain effect for this contrast across groups activated a network of regions including superior parietal and frontal regions (peak cluster: Z=6.19, 40640 voxels, [-34, -56, 44]). There were no significant whole-brain, case-control differences for this contrast. In all participants, the affective bias contrast revealed a positive association with age in two clusters including lateral occipital and lingual gyrus (z=4.01, 4408 voxels, [38, -82, -8]) and frontal orbital, temporal and parahippocampul regions (z=4.13, 1805 voxels, [-20, -2, -22]). The direction of the relationship is the same as that reported in the main text. A group-by-age interaction was also identified located in the precuneous cortex and lingual gyrus (Z=3.83, 733 voxels, [20, -60, 8]), with a stronger association with age seen in the control compared to the depressed group. There was no significant correlation between activation in this contrast and severity of depression in the patient group.

*Retrieval attempt (all retrieval trials > baseline)*

The whole-brain effect for this contrast activated a network including frontal and insular cortices, and paracingulate regions (peak cluster: Z=6.86, 3454 voxels, [-4, 12, 48]). There were no significant whole-brain, case-control differences in the BOLD activation during retrieval attempt and no significant correlations with participant age across groups. Furthermore no group-by-age interaction or correlation with depression severity in the depressed group was observed.

*Supplementary discussion*

When including participants taking antidepressant medications in the analysis (see supplementary materials), differential fMRI findings were evident. The case control difference and group by age interaction for the neural response to memory encoding in addition to the group by age interaction for the neural response to successful retrieval and retrieval attempt no longer reached significance. This could be due to changes in brain activation in this group associated with antidepressant use. Previous literature supports this interpretation, reporting differing neural activation in response to affective images (Rizvi et al., 2013) and memory based tasks (Roy et al, 2010, Walsh et al., 2007) following SSRI administration. In these works, frontal and cingulate regions were highlighted as being implicated in treatment-related effects. Therefore antidepressant medication use could potentially be influencing task-related brain activation for some of the contrasts seen here. During successful encoding for instance, the medicated group showed BOLD responses intermediate between the antidepressant naïve patients and controls (see Figure 2). An alternative interpretation could be the potential effects of increased variance by including these individuals in the analysis. The antidepressant use in this study was part of participants’ clinical treatment and independent from their participation in the wider IMPACT trial, therefore this study is not optimally designed to investigate drug effects. Further research is needed in this area to assess the effect of antidepressant medication on neural activation in adolescents with depression in the context of affective memory and other processes.

**
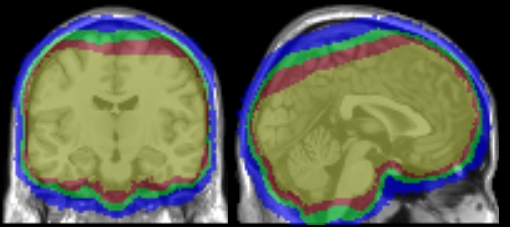
**

**Figure S4:** Brain coverage achieved with the current sample; blue indicates where brain coverage was achieved for 1% of sample, green for 50%, red for 90% and yellow for 100% of sample. Only voxels that have data for all participants were included in the fMRI analyses.
